# Coupling between global gray matter BOLD activity and CSF movement is associated with Aβ and tau burden in preclinical Alzheimer's disease

**DOI:** 10.1002/alz70855_106025

**Published:** 2025-12-24

**Authors:** Gawon Cho, Ryan S O'Dell, Xiao Liu, Helene Benveniste, Daniel Kay, Christopher H van Dyck, Brienne Miner, Adam P Mecca

**Affiliations:** ^1^ Yale School of Medicine, New Haven, CT, USA; ^2^ Alzheimer's Disease Research Unit, Yale University School of Medicine, Department of Psychiatry, New Haven, CT, USA; ^3^ Pennsylvania State University, University Park, PA, USA; ^4^ Yale University, New Haven, CT, USA; ^5^ Brigham Young University, Provo, UT, USA; ^6^ Alzheimer's Disease Research Unit, Yale School of Medicine, New Haven, CT, USA

## Abstract

**Background:**

Reduced glymphatic clearance may contribute to Alzheimer's disease. Low‐frequency CSF flow, which mediates glymphatic clearance, has tight temporal coupling (i.e., anti‐correlation) to global gray matter activity in resting‐state BOLD‐fMRI. But this coupling strength may decrease with AD. Evidence is needed on the association of gray matter BOLD (gBOLD)‐CSF coupling with Aβ, tau, and neurodegeneration (ATN framework) in asymptomatic, preclinical AD.

**Method:**

We studied 21 cognitively normal (CDR=0, MMSE>26) individuals recruited at the Yale Alzheimer's Disease Research Center, who completed structural, resting‐state fMRI (TR/TE=1000ms/30ms; 385 volumes; 2x2x2mm^3^), [^11^C]PiB, [^18^F]MK‐6240, and [^11^C]UCB‐J scans. We calculated Pearson correlation coefficients on the associations of gBOLD‐CSF coupling with global Aβ, tau by ROI corresponding to early Braak stages, and global synaptic density (a potential early marker of neurodegeneration). gBOLD‐CSF coupling was quantified as the cross‐correlation between <0.1Hz gBOLD and CSF signal timeseries at a 4 second lag. gBOLD timeseries were extracted by registering a gray matter mask to rsfMRI scans in native space. CSF timeseries were extracted by averaging signals across CSF voxels in the bottom slice to capture inflow. Global cortical Aβ was quantified as a distribution volume ratio, using a simplified reference tissue model 2 (SRTM2) to generate parametric images of [^11^C]PiB BP_ND_ (reference=whole cerebellum). Tau was quantified for ROIs corresponding with Braak stage 1‐3 as standardized uptake value ratios (SUVR) of [^18^F]MK‐6240 (reference=inferior cerebellar gray matter). Global synaptic density was quantified as [^11^C]UCB‐J BP_ND_generated using SRTM2 (reference=inferior cerebellum).

**Result:**

Mean age, 66.6 (SD=8.9); 57.1% women; 90.5% White; 52.4% *APOE4* carriers. gBOLD‐CSF coupling was negatively associated with tau in entorhinal (r=‐0.49, *p* = 0.025), hippocampal (r=‐0.55, *p* = 0.01), and amygdala ROIs (r=‐0.58, *p* = 0.005). It showed weak negative association with global cortical Aβ (r=‐0.40, *p* = 0.07) and parahippocampal and fusiform tau (r=‐0.40, *p* = 0.07; r=‐0.38, *p* = 0.085). gBOLD‐CSF coupling was not associated with lingual tau and global synaptic density (*p*s>0.05).

**Conclusion:**

Contrary to our expectations, individuals with higher Aβ/tau showed stronger gBOLD‐CSF anti‐correlation. Reasons remain unclear, but it is possible that individuals with ATN were more likely to sleep during rsfMRI, leading to increased fluid movement. Our findings suggest the importance of adjusting for sleep‐wake states when imaging brain fluid transport.

